# Enhancement of the International prognostic index with β2-microglobulin, platelet count and red blood cell distribution width: a new prognostic model for diffuse large B-cell lymphoma in the rituximab era

**DOI:** 10.1186/s12885-022-09693-z

**Published:** 2022-05-27

**Authors:** Haizhu Chen, Qiaofeng Zhong, Yu Zhou, Yan Qin, Jianliang Yang, Peng Liu, Xiaohui He, Shengyu Zhou, Changgong Zhang, Lin Gui, Sheng Yang, Liqiang Zhou, Yuankai Shi

**Affiliations:** Department of Medical Oncology, National Cancer Center/National Clinical Research Center for Cancer/Cancer Hospital, Chinese Academy of Medical Sciences & Peking Union Medical College, Beijing Key Laboratory of Clinical Study on Anticancer Molecular Targeted Drugs, No. 17 Panjiayuan Nanli, Chaoyang District, Beijing, 100021 China

**Keywords:** Diffuse large B-cell lymphoma, β2-microglobulin, Platelet count, Red blood cell distribution width, Prognosis, International prognostic index

## Abstract

**Background:**

This study aimed to propose a new user-friendly, cost effective and robust risk model to facilitate risk stratification for diffuse large B-cell lymphoma (DLBCL) treated with frontline R-CHOP regimens.

**Methods:**

Data on 998 patients with de novo DLBCL diagnosed between Jan 1st, 2005 and Dec 31st, 2018 at our center, who received frontline R-CHOP or R-CHOP-like regimens, were retrospectively collected. Patients were randomly divided into the training cohort (*n* = 701) and the validation cohort (*n* = 297). A new prognostic model for overall survival (OS) was built based on the training cohort. The performance of the new model was compared with International prognostic index (IPI), revised IPI (R-IPI) and National Comprehensive Cancer Network (NCCN)-IPI (NCCN-IPI). The new model was validated in the validation cohort.

**Results:**

The multivariate analysis of the training cohort showed that the IPI, β2-microglobulin, platelet count and red blood cell distribution width were independent factors for OS, which were incorporated into the new prognostic model. Patients were stratified into low risk, low-intermediate risk, high-intermediate risk, high risk and very high risk groups, with distinct survival outcomes. The new model achieved good C-indexes for 5-year OS prediction of 0.750 (95%CI 0.719–0.781) and 0.733 (95%CI 0.682–0.784) in the training and validation cohorts, respectively, and displayed well-fitted calibration curves. The C-index and the time-dependent ROC analysis demonstrated better performance of the new model than the IPI, R-IPI and NCCN-IPI in both training and validation cohorts. The integrated Brier score for predicting 5-year OS of the new model was lower than that of the IPI, R-IPI and NCCN-IPI in both cohorts, and decision curve analysis also showed a higher net benefit, indicating the superiority of the new model over the conventional models.

**Conclusion:**

The new prognostic model might be a useful predictive tool for DLBCL treated with R-CHOP regimens. Further external validation is warranted.

**Supplementary Information:**

The online version contains supplementary material available at 10.1186/s12885-022-09693-z.

## Introduction

Diffuse large B-cell lymphoma (DLBCL), the most frequent subtype of non-Hodgkin’s lymphoma (NHL), is a markedly heterogeneous disease with varying clinical course and outcomes [1]. The addition of rituximab to the conventional CHOP (cyclophosphamide, doxorubicin, vincristine and prednisone) regimens has significantly extended the survival of patients with DLBCL [2, 3]. Despite the remarkable improvement, attempts to improve outcomes of patients who cannot be cured by this standard first-line therapy remain challenging. Therefore, there is an unmet need to develop an accurate risk classification and identify high-risk patients.

The International Prognostic Index (IPI), which was developed in the pre-rituximab era, identified four discrete risk groups, and became a well-established prognostic tool for aggressive NHL [4]. Despite that the IPI remains valid in the era of immunochemotherapy, its ability to distinguish between the previous four risk groups has diminished [5, 6]. Other efforts to improve the prognostic power of IPI included the redistribution of the conventional IPI score (revised IPI, R-IPI) [5] and development of an enhanced National Comprehensive Cancer Network (NCCN)-IPI [7]. These two scoring systems were reported to provide better prognostic guidance, but still failing to identify extremely high-risk patient subgroups [8–10].

There is emerging evidence that biomarkers for tumor microenvironment and host immunity may confer prognostic information. Several hematologic markers, including serum β2-microglobulin (β2M) [9, 11, 12], albumin [13, 14] and components derived from baseline complete blood cell counts [11, 15–20] have been proved to be of prognostic value in DLBCL. New prognostic models that focused on integrating these new factors into traditional variables were developed, enabling to distinguish a higher risk group compared with the IPI or NCCN-IPI [9, 16, 21]. Although promising, some of these variables or models have yet to be further externally validated. Besides, all these models were developed based upon patients derived from western cohorts, and there are currently no data to confirm them in Chinese DLBCL patients.

This study aimed to identify prognostic factors, especially analyze the prognostic value of hematologic parameters in a large cohort of Chinese DLBCL patients treated with R-CHOP or R-CHOP-like regimens. We attempted to propose a new user-friendly, cost effective and robust risk model to facilitate risk stratification for this disease.

## Materials and methods

### Patient cohort

Patients with de novo DLBCL diagnosed between Jan 1st, 2005 and Dec 31st, 2018 at our hospital were retrospectively reviewed. The criteria for inclusion included: (1) histologically confirmed diagnosis of DLBCL according to the WHO classification [22]; (2) patients who received frontline therapy with R-CHOP or R-CHOP-like regimens with curative intent; (3) patients with the complete clinical data required for different analyses; (4) patients with complete treatment and follow-up information. All histological subtypes of DLBCL were eligible, except for primary central nervous system DLBCL due to its special biological features compared to other DLBCL types. Patients receiving treatment with non-curative intent or chemotherapy with lower dose, such as R-mini-CHOP, were not eligible. Patients with missing laboratory data were excluded. Patients with positive human immunodeficiency virus were also ineligible. Since patients were excluded on the grounds of missing data, which were at random and without knowledge of outcomes, there was no intentional selection bias. A total of 998 eligible patients were ultimately included in the current study, and were randomly divided into the training cohort (*n* = 701) and the validation cohort (*n* = 297) according to a ratio of 7:3.

The baseline clinical features included age, gender, Eastern Cooperative Oncology Group (ECOG) performance status (PS), number of extranodal disease sites, lactate dehydrogenase (LDH), β2M, serum creatinine, albumin, Ann Arbor stage, bone marrow (BM) involvement, IPI, R-IPI, NCCN-IPI, and complete blood count (CBC) parameters. Treatment, treatment response and follow-up data were also collected. LDH, β2M, serum creatinine, and albumin were obtained from blood biochemical profiles which were measured by an automated biochemical analyzer (Roche Cobas C8000, Germany) using standard methods. CBC variables consisted of absolute lymphocyte count (ALC), absolute monocyte count (AMC), absolute neutrophil count (ANC), platelet (PLT), hemoglobin, red blood cell distribution width (RDW), platelet distribution width (PDW) and mean platelet volume (MPV). These CBC parameters were obtained and calculated by a standard automated complete blood analyzer (Sysmex XN-9000, Japan) at initial diagnosis. Regarding RDW values in our study, coefficient variation of red blood cell volume distribution width (RDW-CV) was used, rather than standard deviation in red cell distribution width (RDW-SD). The normal reference for RDW-CV ranged between 11.6 and 14.6% in our hospital. The lymphocyte to monocyte ratio (LMR), the neutrophil to lymphocyte ratio (NLR) and the platelet to lymphocyte ratio (PLR) were calculated.

### Treatment evaluation and outcomes

All patients received frontline standard R-CHOP or R-CHOP like regimens with curative intent. Radiotherapy was administrated following chemotherapy for residual disease or previous bulky disease as consolidation therapy. Treatment response was evaluated according to the International Working Group criteria [23].

The primary endpoint was overall survival (OS), defined as the initial diagnosis until death from any cause or last follow-up. Progression-free survival (PFS) was defined as the initial diagnosis until the first disease progression, relapse or death from any cause, whichever came first, or last follow-up.

### Statistical analysis

Continuous variables were compared using the Mann–Whitney U analysis, and categorical variables were compared with the Chi-square or Fisher’s exact test. The optimal cutoff values of ALC, AMC, ANC, PLT, RDW, PDW, MPV, LMR, NLR and PLR for predicting OS in the training cohort were determined using the Maximally Selected Rank Statistics in R software environment [24]. As a result, the optimal cutoff points of ALC, AMC, ANC, PLT, RDW, PDW, MPV, LMR, NLR and PLR were 1.75 × 10^9^/L, 0.65 × 10^9^/L, 6.41 × 10^9^/L, 157 × 10^9^/L, 14.5%, 12.8 fl, 9.1 fl, 2.55, 3.68 and 183.7, respectively. By contrast, the cutoff values of 35 g/L and 120 g/L for albumin and hemoglobin concentrations, respectively, were selected according to previous studies [14, 25]. OS and PFS were estimated using the Kaplan-Meier method, and compared by the log rank test. The univariate and multivariate analyses were performed by the Cox proportional hazards regression model.

The training cohort was used to establish the new prognostic model for OS, and validation of the new model was carried out using the validation cohort. All variables with prognostic significance identified in univariate analysis of the training cohort were included for stepwise multivariate Cox regression analysis. A final model was formulated based on the results of multivariate analysis. The Harrell’s concordance index (C-index), the time-dependent receiver operating characteristic (ROC) and corresponding area under curve (AUC), as well as calibration with 1000 bootstrap samples were applied to evaluate the predictive performance of the new model [26]. Additionally, the cumulative prediction errors or integrated Brier score (IBS) were calculated to evaluate the predictive ability of prognostic models over time [27]. Decision curve analysis (DCA) was applied to assess the utility of models for clinical decision making [28]. All statistical analyses were conducted using IBM SPSS Statistics, Version 26.0 and packages of “maxstat”, “Hmisc”, “rms”, “survival”, “time ROC”, “pec” and “ggDCA” packages in R, version 3.6.2 (http://www.r-project.org/). The two-sided *P*-values < 0.05 were determined to be statistically significant.

## Results

### Patient characteristics and survival

A total of 998 eligible patients were enrolled, with 701 and 297 patients divided into the training and validation cohorts, respectively. For all patients, median age was 53 (range, 7–83) years, and more than half (55.2%) of the patients were male. The majority of patients (89.3%) had an ECOG PS of 0–1, and 61.4% of cases presented with Ann Arbor stage I/II disease. The baseline features were comparable between the training cohort and the validation cohort (Table 1).

**Table 1:** Baseline patient characteristics

Characteristic	Overall cohort (*n* = 998)	Training cohort (*n* = 701)	Validation cohort (*n* = 297)	*P*
	N(%)	N(%)	N(%)	
Age, years				
Median (range)	53 (7–83)	53 (7–83)	54 (15–81)	0.847
≤ 60	653 (65.4)	460 (65.6)	193 (65.0)	
> 60	345 (34.6)	241 (34.4)	104 (35.0)	
Gender				
Male	551 (55.2)	388 (55.3)	163 (54.9)	0.892
Female	447 (44.8)	313 (44.7)	134 (45.1)	
ECOG PS				
0–1	891 (89.3)	623 (88.9)	268 (90.2)	0.525
≥ 2	107 (10.7)	78 (11.1)	29 (9.8)	
Ann Arbor stage				
I	232 (23.2)	172 (24.5)	60 (20.2)	0.126
II	381 (38.2)	256 (36.5)	125 (42.1)	
III	156 (15.6)	104 (14.8)	52 (17.5)	
IV	229 (22.9)	169 (24.1)	60 (20.2)	
Number of extranodal sites				
< 2	755 (75.7)	529 (75.5)	226 (76.1)	0.832
≥ 2	243 (24.3)	172 (24.5)	71 (23.9)	
Bone marrow involvement				
Yes	56 (5.6)	42 (6.0)	14 (4.7)	0.423
No	942 (94.4)	659 (94.0)	283 (95.3)	
Lactate dehydrogenase level				
Elevated	457 (45.8)	330 (47.1)	127 (42.8)	0.211
Normal	541 (54.2)	371 (52.9)	170 (57.2)	
β2-microglobulin level				
Elevated	310 (31.1)	222 (31.7)	88 (29.6)	0.524
Normal	688 (68.9)	479 (68.3)	209 (70.4)	
Serum creatinine level				
Elevated	28 (2.8)	22 (3.1)	6 (2.0)	0.328
Normal	970 (97.2)	679 (96.9)	291 (98.0)	
IPI risk group (score)				
Low (0–1)	552 (55.3)	390 (55.6)	162 (54.5)	0.177
Low-intermediate (2)	203 (20.3)	131 (18.7)	72 (24.2)	
High-intermediate (3)	156 (15.6)	116 (16.5)	40 (13.5)	
High (4–5)	87 (8.7)	64 (9.1)	23 (7.7)	
R-IPI risk group (score)				
Very good (0)	251 (25.2)	174 (24.8)	77 (25.9)	0.320
Good (1–2)	504 (50.5)	347 (49.5)	157 (52.9)	
Poor (3–5)	243 (24.3)	180 (25.7)	63 (21.2)	
NCCN-IPI risk group (score)				
Low (0–1)	316 (31.7)	221 (31.5)	95 (32.0)	0.525
Low-intermediate (2–3)	456 (45.7)	313 (44.7)	143 (48.1)	
High-intermediate (4–5)	199 (19.9)	146 (20.8)	53 (17.8)	
High (≥6)	27 (2.7)	21 (3.0)	6 (2.0)	
Hemoglobin (g/L)				
≥ 120	780 (78.2)	553 (78.9)	227 (76.4)	0.391
< 120	218 (21.8)	148 (21.1)	70 (23.6)	
Albumin (g/L)				
≥ 35	931 (93.3)	648 (92.4)	283 (95.3)	0.100
< 35	67 (6.7)	53 (7.6)	14 (4.7)	

*Abbreviations*: *ECOG* Eastern Cooperative Oncology Group, *PS* Performance status, *IPI* International Prognostic Index, *R-IPI* Revised International Prognostic Index, *NCCN-IPI* National Comprehensive Cancer Network International Prognostic Index

The median follow-up duration of the training and validation cohorts were 85.2 (range, 0.5–179.6) months and 86.4 (range, 0.5–157.3) months, respectively. During the follow-up, 269 and 106 events for PFS occurred in the training and validation cohorts, respectively. Besides, 207 deaths were observed in the training cohort, with 85 deaths in the validation cohort.

### Construction of the new prognostic model for overall survival

Univariate analysis of the training cohort showed that the IPI factors (age, ECOG PS, Ann Arbor stage, number of extranodal disease sites and LDH), the status of BM involvement, β2M, serum creatinine, albumin and most CBC variables were significantly associated with PFS and OS (Supplementary Table S1). The PFS and OS according to β2M, RDW and PLT were displayed in Supplementary Fig. S1. Of note, the IPI score as a whole, rather than single prognostic indicators, was incorporated into further multivariate analysis. Multivariate analysis showed that besides the IPI score, elevated β2M level (HR 1.411, 95%CI 1.040–1.913, *P* = 0.027), PLT < 157 × 10^9^/L (HR 1.548, 95%CI 1.038–2.308, *P* = 0.032) and RDW ≥14.5% (HR 1.758, 95%CI 1.214–2.547, *P* = 0.003) were significantly associated with inferior OS (Supplementary Table S2). Regarding PFS, the IPI, β2M, PLT and RDW remained independent predictors.

**Fig. 1:**
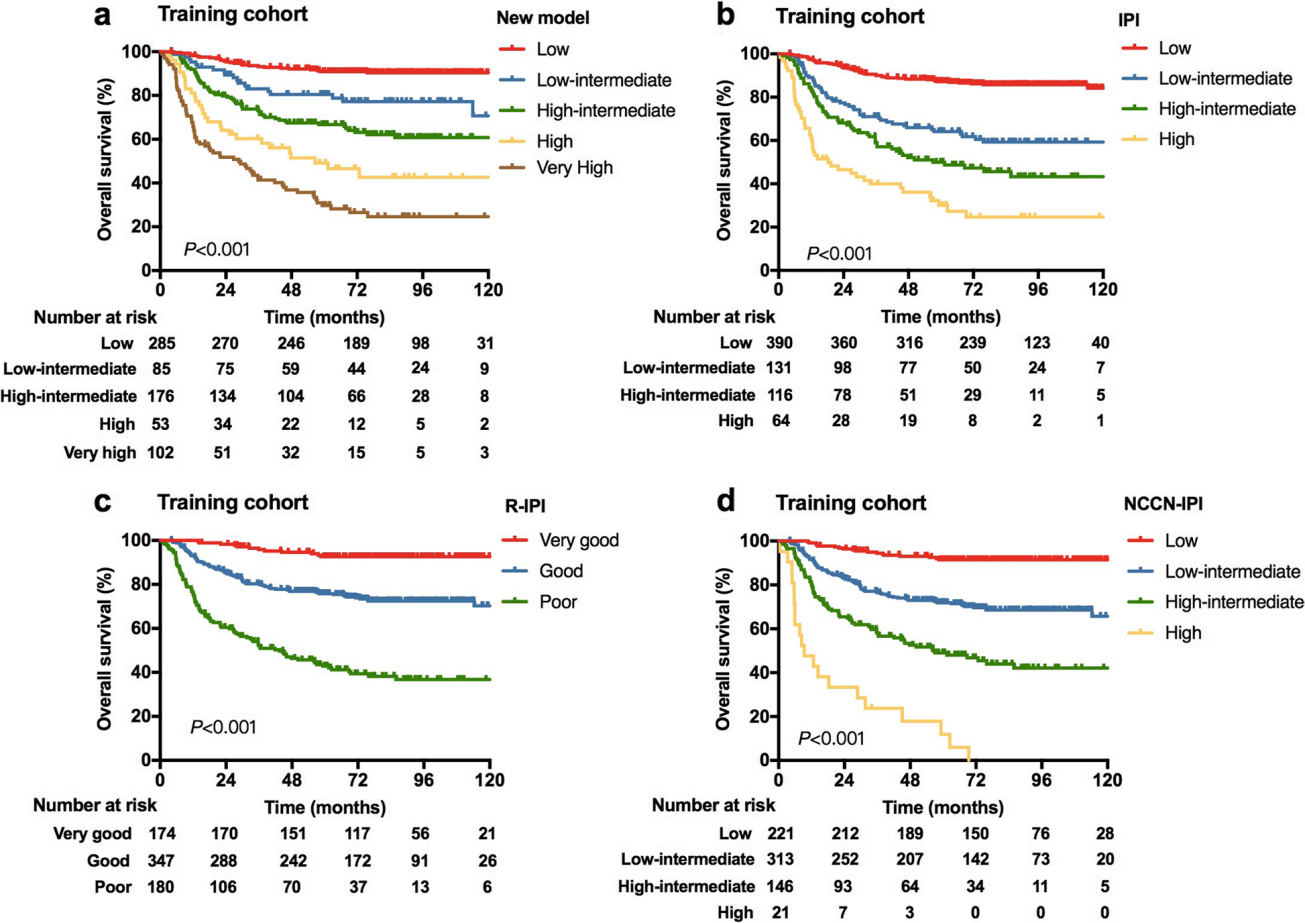
Overall survival (OS) for risk groups defined by four prognostic models in the training cohort (*n* = 701). **a** OS stratified by the new model; (**b**) OS stratified by the International Prognostic Index (IPI); (**c**) OS stratified by the revised IPI (R-IPI); (**d**) OS stratified by the National Comprehensive Cancer Network-IPI (NCCN-IPI)

Based on the corresponding HRs of the prognostic factors derived from the multivariate analysis for OS, a new prognostic model was constructed. The scoring point assigned to each prognostic factor was in the following way: IPI (low-intermediate risk group, two points; high-intermediate risk group, three points; high risk group, five points), elevated β2M level, PLT < 157 × 10^9^/L and RDW ≥14.5%, with one point each for the last three risk factors (Table 2). As a result, the new model scored a maximum of eight points. Patients in the training cohort were stratified into five distinct risk groups: 285 (40.7%) patients as low risk (0 point), 85 (12.1%) as low-intermediate risk (1 point), 176 (25.1%) as high-intermediate risk (2–3 points), 53 (7.6%) as high risk (4 points) and 102 (14.6%) as very high risk (≥5 points), with the 5-year OS rates of 90.9, 80.4, 66.7, 49.1 and 29.7%, respectively (*P* < 0.001) (Table 3 and Fig. 1a). The new model demonstrated favorable accuracy in predicting OS, with a C-index for 5-year OS prediction of 0.750 (95%CI 0.719–0.781). The calibration plots for predicting the probability of survival at 5 years also graphically showed good agreement between the prediction by the new model and actual prediction (Supplementary Fig. S2a). Similar results were observed for PFS, and the new model could also distinguish patients with distinct PFS (Table 3 and Supplementary Fig. S3a).

**Table 2:** Independent factors of progression-free survival and overall survival from multivariate analysis of the training cohort

Characteristic	Progression-free survival		Overall survival		Score
	HR (95%CI)	*P*	HR (95%CI)	*P*	
IPI risk group					
Low (0–1)	Reference		Reference		0
Low-intermediate (2)	2.003 (1.411–2.845)	< 0.001	2.401 (1.593–3.618)	< 0.001	2
High-intermediate (3)	2.922 (2.053–4.159)	< 0.001	3.346 (2.211–5.064)	< 0.001	3
High (4–5)	4.073 (2.684–6.182)	< 0.001	5.341 (3.301–8.644)	< 0.001	5
β2-microglobulin level					
Normal	Reference		Reference		0
Elevated	1.543 (1.181–2.016)	0.001	1.411 (1.040–1.913)	0.027	1
PLT (×10^9^/L)					
≥ 157	Reference		Reference		0
< 157	1.433 (1.010–2.034)	0.044	1.548 (1.038–2.308)	0.032	1
RDW (%)					
< 14.5	Reference		Reference		0
≥ 14.5	1.438 (1.022–2.023)	0.037	1.758 (1.214–2.547)	0.003	1

*Abbreviations*: *IPI* International Prognostic Index, *HR* Hazard ratio, *PLT* Platelet, *RDW* Red blood cell distribution width

This multivariate analysis included the grouped IPI but excluded individual IPI factors

**Table 3:** Comparison of the new prognostic model with conventional models for stratifying survival outcomes in the training and validation cohorts

Risk group (score)	Training cohort (*n* = 701)			Validation cohort (*n* = 297)		
	n (%)	5-y PFS, % (95%CI)	5-y OS,% (95%CI)	n (%)	5-y PFS, % (95%CI)	5-y OS, % (95%CI)
New model						
Low (0)	285 (40.7)	84.0 (79.8–88.4)	90.9 (87.6–94.7)	118 (39.7)	87.2 (81.3–93.5)	91.2 (86.2–96.6)
Low-intermediate (1)	85 (12.1)	72.1 (62.9–82.5)	80.4 (72.2–89.5)	38 (12.8)	68.0 (54.5–84.8)	77.8 (65.3–92.8)
High-intermediate (2–3)	176 (25.1)	55.7 (48.8–63.6)	66.7 (59.9–74.3)	89 (30.0)	56.7 (47.3–68.1)	65.6 (56.3–76.4)
High (4)	53 (7.6)	33.7 (23.0–49.2)	49.1 (36.9–65.2)	22 (7.4)	36.4 (20.9–63.2)	47.7 (30.5–74.7)
Very High (≥5)	102 (14.6)	19.0 (12.5–29.1)	29.7 (21.7–40.6)	30 (10.1)	23.3 (12.2–44.6)	26.7 (14.7–48.3)
IPI						
Low (0–1)	390 (55.6)	80.0 (76.1–84.1)	87.2 (83.9–90.7)	162 (54.5)	80.5 (74.6–86.9)	87.1 (82.0–92.6)
Low-intermediate (2)	131 (18.7)	55.3 (47.3–64.5)	64.1 (56.2–73.1)	72 (24.2)	56.9 (46.5–69.6)	64.7 (54.5–76.9)
High-intermediate (3)	116 (16.5)	33.7 (26.0–43.7)	50.0 (41.4–60.3)	40 (13.5)	47.5 (34.3–65.8)	55.0 (41.6–72.8)
High (4–5)	64 (9.1)	20.6 (12.4–34.3)	30.1 (20.3–44.6)	23 (7.7)	17.4 (7.1–42.4)	21.7 (10.0–47.2)
R-IPI						
Very good (0)	174 (24.8)	87.1 (82.2–92.3)	92.6 (88.7–96.7)	77 (25.9)	81.7 (73.4–90.8)	86.5 (79.0–94.7)
Good (1–2)	347 (49.5)	67.1 (62.3–72.2)	75.8 (71.4–80.6)	157 (52.9)	69.1 (62.2–76.8)	77.2 (70.8–84.1)
Poor (≥3)	180 (25.7)	29.1 (23.1–36.8)	42.8 (35.9–51.1)	63 (21.2)	36.5 (26.4–50.6)	42.9 (32.2–57.0)
NCCN-IPI						
Low (0–1)	221 (31.5)	85.3 (80.7–90.1)	91.5 (87.8–95.3)	95 (32.0)	80.8 (73.3–89.2)	85.9 (79.0–93.3)
Low-intermediate (2–3)	313 (44.7)	63.8 (58.6–69.4)	71.9 (66.9–77.1)	143 (48.1)	67.5 (60.2–75.7)	75.7 (69.0–83.2)
High-intermediate (4–5)	146 (20.8)	31.5 (24.7–40.1)	49.0 (41.3–58.3)	53 (17.8)	37.6 (26.5–53.2)	44.0 (32.4–59.9)
High (≥6)	21 (3.0)	9.4 (1.7–51.1)	11.9 (3.4–41.3)	6 (2.0)	16.7 (2.8–99.7)	16.7 (2.8–99.7)

*Abbreviations*: *PFS* Progression-free survival, *OS* Overall survival, *5-y* 5-year, *CI* Confidence interval, *IPI* International Prognostic Index, *R-IPI* Revised International Prognostic Index, *NCCN-IPI* National Comprehensive Cancer Network International Prognostic Index

### Validation of the new prognostic model

**Fig. 2:**
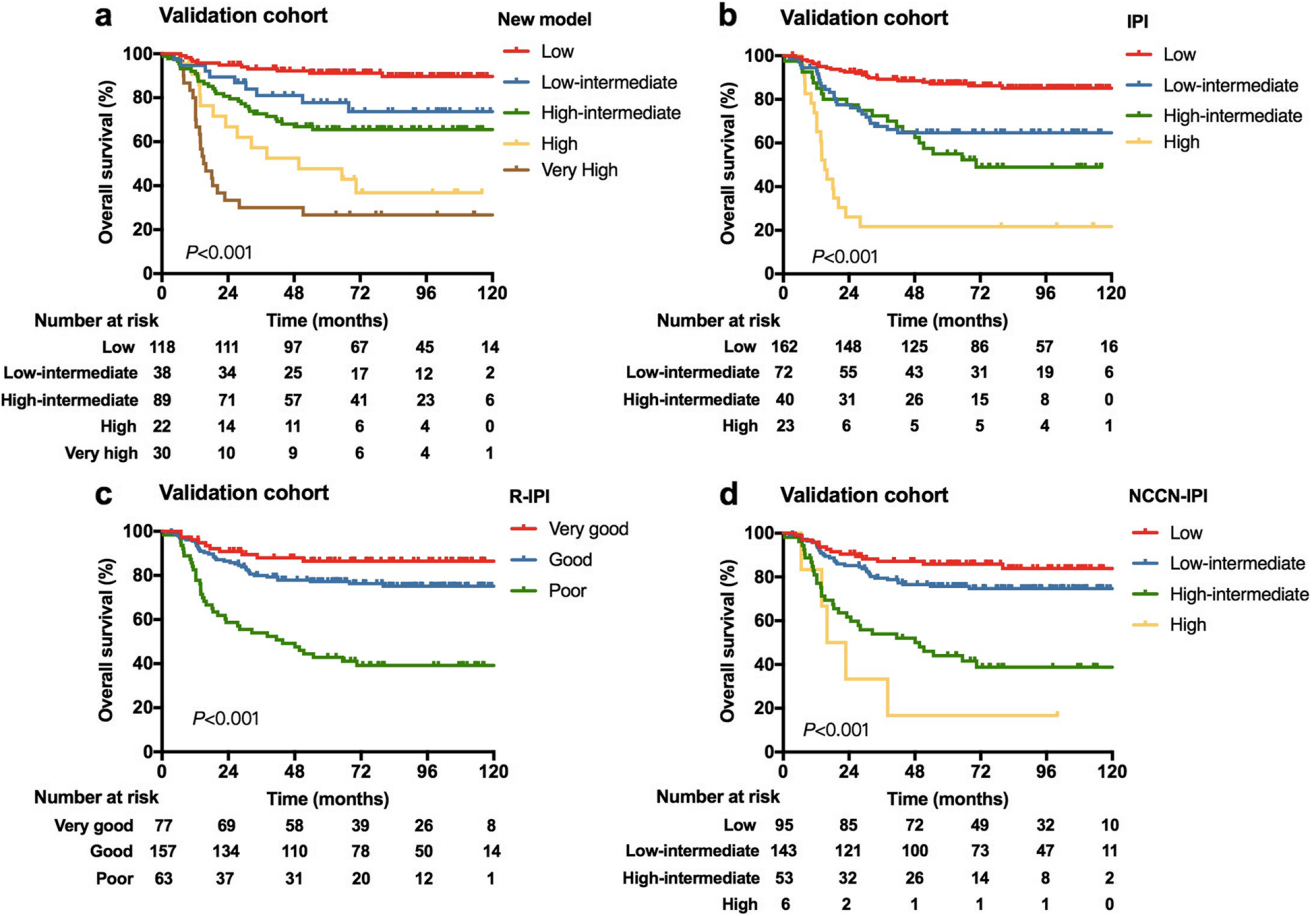
Overall survival (OS) for risk groups defined by four prognostic models in the validation cohort (*n* = 297). **a** OS stratified by the new model; (**b**) OS stratified by the International Prognostic Index (IPI); (**c**) OS stratified by the revised IPI (R-IPI); (**d**) OS stratified by the National Comprehensive Cancer Network- IPI (NCCN-IPI)

The new model applied to the validation cohort also separated patients into five risk groups with distinct survival outcomes, with 118 (39.7%) patients in low risk, 38 (12.8%) in low-intermediate risk, 89 (30.0%) in high-intermediate risk, 22 (7.4%) in high risk and 30 (10.1%) in very high risk group. Patients in corresponding risk groups had 5-year OS rates of 91.2, 77.8, 65.6, 47.7 and 26.7% (*P* < 0.001), and the 5-year PFS rates of 87.2, 68.0, 56.7, 36.4 and 23.3%, respectively (*P* < 0.001) (Table 3 and Fig. 2a). In the validation cohort, the new model displayed favorable discriminative ability, with a C index of 0.733 (95%CI 0.682–0.784) for predicting 5-year OS. Also, there was a good calibration curve for the prediction of 5-year OS (Supplementary Fig. S2b).

### Comparison of the new model with conventional prognostic models

Based on Kaplan Meier curves, the new model seemed to display better discrimination of OS compared with the IPI, R-IPI and NCCN-IPI (Table 3, Figs. 1 and 2). Comparing with the original IPI, the new model identified a subgroup of patients with superior survival outcomes, with 5-year OS rates in the low risk group from the training cohort of 90.9% vs. 87.2%, and that from the validation cohort of 91.2% vs. 87.1%. Besides, the new model identified a higher proportion of patients with poor prognosis comparing with IPI. The R-IPI score also distinguished a subgroup of patients with favorable OS, whereas patients with high risk could not be well discriminated. With the new model, the very high risk group had inferior 5-year OS rates than those classified as the poor group by R-IPI in both training cohort (29.7% vs. 42.8%) and validation cohort (26.7% vs 42.9%). Additionally, the NCCN-IPI differentiated patients with favorable OS, and also displayed a good ability to identify a subgroup of patients with very poor survival (5-year OS rate, 11.9 and 16.7% for training and validation cohorts, respectively). However, the proportion of high risk group with NCCN-IPI was small in our patient cohort, with only 3.0 and 2.0% of patients classified as high risk in the training cohort and validation cohort, respectively. Similar results were observed for PFS, and the new model could also discriminate PFS better than the IPI, R-IPI and NCCN-IPI (Table 3, Supplementary Figs. S3 and S4).

**Fig. 3:**
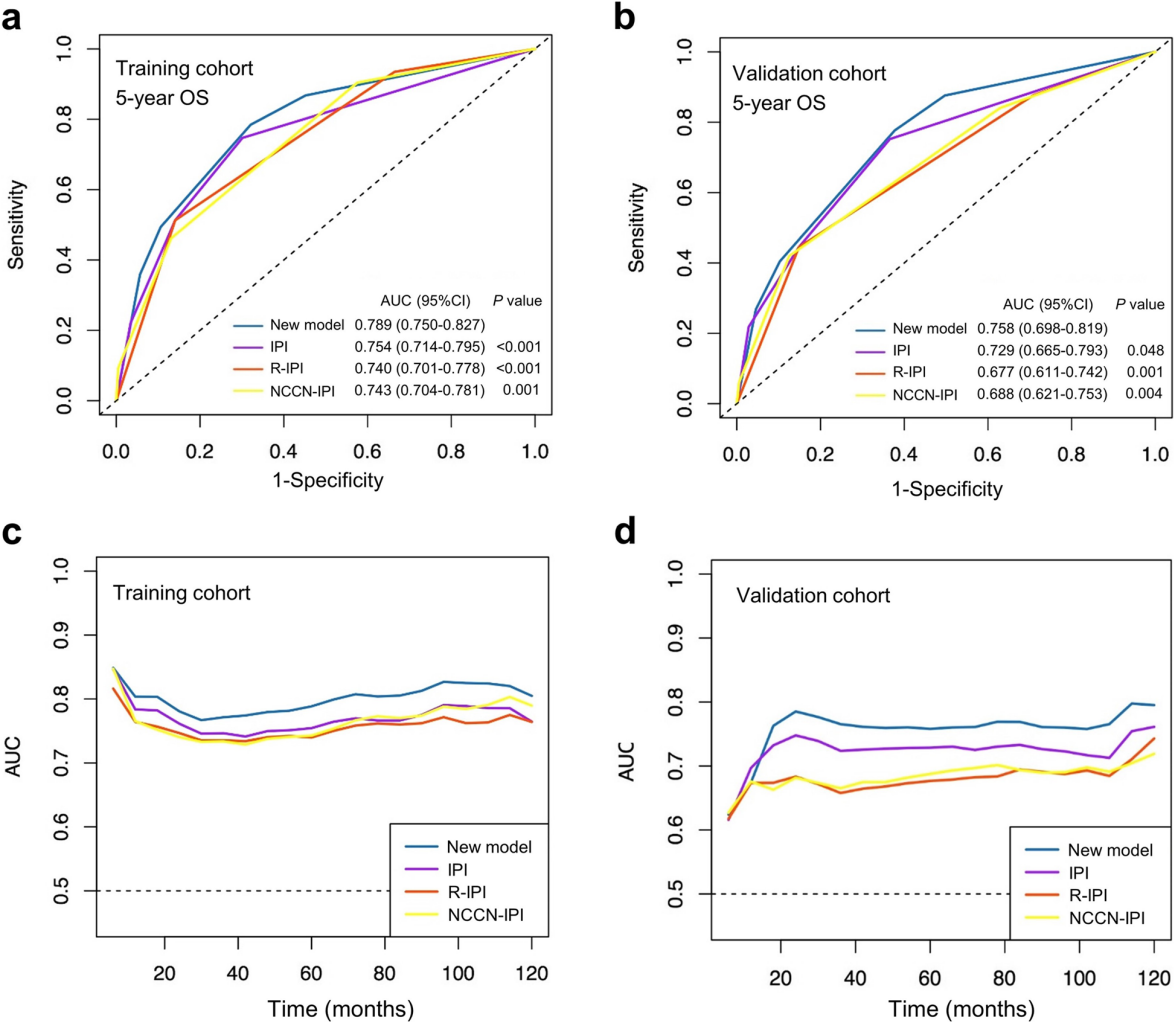
Comparison of the predictive performance between the new model and the conventional prognostic models. **a** The area under curve (AUC) for 5-year overall survival (OS) prediction of the four prognostic models (the new prognostic model, IPI, R-IPI and NCCN-IPI) in the training cohort; (**b**) The AUC for 5-year OS prediction of the four prognostic models in the validation cohort; (**c**) The time-dependent AUC of the four prognostic models for predicting OS between 6 and 120 months in the training cohort; (**d**) The time-dependent AUC of the four prognostic models for predicting OS between 6 and 120 months in the validation cohort. IPI, International Prognostic Index; R-IPI, revised International Prognostic Index; NCCN-IPI, National Comprehensive Cancer Network International Prognostic Index

In the training cohort, ROC analysis showed that the AUC of the new model for predicting 5-year OS was 0.789, which was significantly higher than that of the IPI (0.754; *P* < 0.001), R-IPI (0.740; *P* < 0.001) and NCCN-IPI (0.743; *P* = 0.001) (Fig. 3a). In the validation cohort, the AUC of the new model (0.758) for predicting the 5-year OS was also significantly higher than that of the IPI (0.729; *P* = 0.048), R-IPI (0.667; *P* = 0.001) and NCCN-IPI (0.688; *P* = 0.004) (Fig. 3b). Importantly, the AUC of the new model for OS prediction at specific time points (6 months to 10 years) was consistently higher than that of conventional prognostic models in both training and validation cohorts (Fig. 3c-d). Moreover, the C-index of the new model for predicting 5-year OS was also higher than that of conventional prognostic models in both cohorts (Supplementary Table S3), indicating that the new model displays better accuracy.

**Fig. 4:**
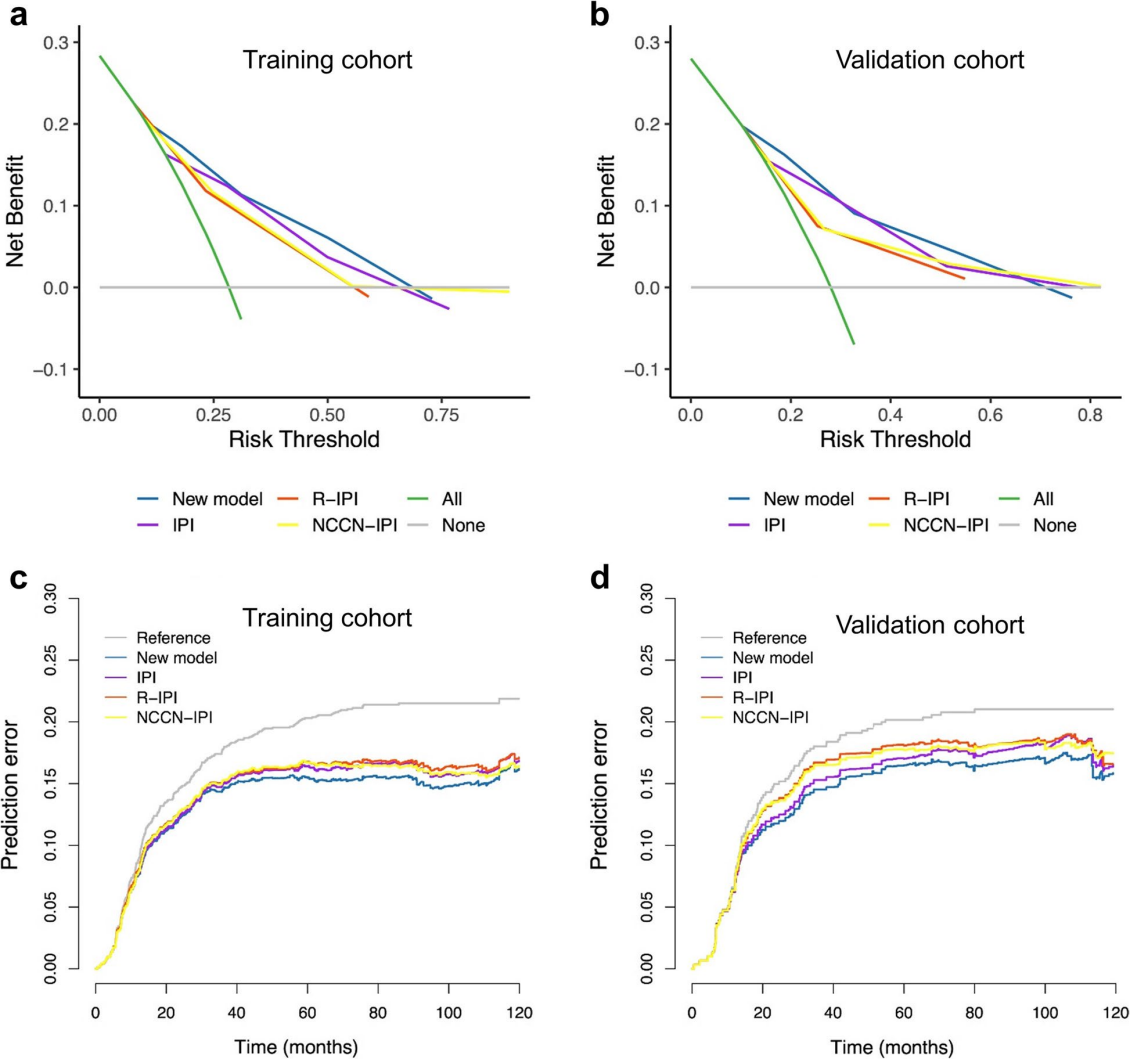
Decision curve analysis and prediction error curves. **a** Decision curve analysis (DCA) for predicting 5-year overall survival (OS) in the training cohort; (**b**) DCA for predicting 5-year OS in the validation cohort; (**c**) Prediction error curves for 5-year OS prediction of four prognostic models in the training cohort; (**d**) Prediction error curves for 5-year OS prediction of four prognostic models in the validation cohort. Note: In Fig. 4a and b, the horizontal solid grey line represents the assumption that no patients would be dead, and the solid green line represents the assumption that all patients would be dead. The solid blue, purple, red and yellow lines indicate the net benefit using the new model, IPI, R-IPI and NCCN-IPI, respectively. In Fig. 4c and d, the grey curve represents a default benchmark Kaplan-Meier model, and the blue, purple, red and yellow curves represent the new model, IPI, R-IPI and NCCN-IPI, respectively. IPI, International Prognostic Index; R-IPI, revised International Prognostic Index; NCCN-IPI, National Comprehensive Cancer Network International Prognostic Index

Furthermore, the new model showed a higher net benefit compared to the IPI, R-IPI and NCCN-IPI at most threshold probabilities, ensuring to achieve maximum clinical benefit (Fig. 4a-b). Overall, the DCA curve indicated that the new model was profitable for making valuable clinical decision. The predictive performance of these models was further measured by the cumulative prediction errors based on IBS. In the training cohort, the IBS for the 5-year OS prediction of the new model was 0.116, which was lower than that of the IPI (0.119), R-IPI (0.121) and NCCN-IPI (0.121). Similarly, compared with the new model (0.114), the IPI (0.119), R-IPI (0.128) and NCCN-IPI (0.127) showed higher IBS in the validation cohort. The prediction error curves for each model were presented in Fig. 4c-d.

## Discussion

In the present study, besides the IPI score, three risk factors including baseline β2M, PLT and RDW, were independently predictive of OS. A new prognostic model, comprising the IPI and these three factors, was developed for newly diagnosed DLBCL patients treated with R-CHOP or R-CHOP-like regimens. The new model performed well in predicting OS, and stratified patients into five risk groups with distinct survival outcomes. When compared with the conventional IPI, R-IPI and NCCN-IPI, the new model exhibited better accuracy and discrimination for survival outcome prediction.

During the past decades, the ability of IPI, R-IPI and NCCN-IPI to identify a subgroup of patients with very dismal survival has been proved to be unsatisfactory. Gene expression profiling [29, 30], prognostic biomarkers based on immunohistochemistry [31, 32], mutational analyses [33–35] and novel molecular subtypes [36–38] have provided crucially predictive information in DLBCL, allowing for better individual risk prediction. Unfortunately, although with significant prognostic relevance, many of these methods are costly, cumbersome and technically challenging or lack reproducibility, thus they are not yet widely implemented in clinical practice to date. More efforts are needed to develop more simple and valuable prognostic tools for accurate risk stratification.

Given the need for accurate prognostic factors, previous studies also have attempted to investigate the prognostic impact of hematologic markers on DLBCL, and several prognostic indices have been identified. β2M, which forms the light chain subunit of histocompatibility complex class I antigens, might reflect the intrinsic biological feature of the tumor to some extent [39]. Considerable attention has been paid for the observation that elevated β2M level was a predictor of poor survival in both the pre- and post-rituximab era, and several prognostic models based upon β2M were proposed [9, 11, 12]. One previous study involving a large series of patients with DLBCL in Spain developed a novel scoring system, the GELTAMO-IPI, based on the incorporation of β2M into the NCCN-IPI variables [9]. The GELTAMO-IPI had higher accuracy than the NCCN-IPI, and conferred an advantage in identifying an authentic high-risk group. Although the prognostic value of β2M in DLBCL has been reproducibly confirmed, the mechanism underlying this has yet to be fully elucidated. One explanation was that β2M might be related to total tumor burden and cell turnover rate [40]. Other explanations included that β2M could be associated with other biological features that accounted for the functional regulation of growth, apoptosis, and metastasis of cancer cells [41]. Notably, β2M is excreted mainly via the kidneys, thus renal failure may lead to an elevation of serum β2M level. In our study, the association of serum β2M level with serum creatinine was assessed. As expected, patients with elevated serum creatinine level were more likely to have elevated serum β2M level (*P* < 0.001) (Supplementary Table S4). After adjustment for serum creatinine level in the multivariate analysis, elevated β2M remained strongly predictive of inferior survival. Our result was consistent with a previous study in which impaired renal function was positively associated with elevated serum β2M level [42]. In that study, elevated β2M remained an independent adverse prognostic factor for PFS and exhibited a strong trend of association with inferior OS after controlling for impaired renal function. Subgroup analysis of that study indicated elevated β2M was significantly associated with worse survival in patients with normal renal function, and also reflected poor prognosis even in patients with impaired renal function [42]. Given these findings, despite that renal failure can increase serum β2M level, the prognostic impact of β2M in DLBCL may be not influenced by renal function. Serum β2M may serve as a strong prognostic marker in DLBCL.

With a growing body of evidence on the role of host immunity and the tumor microenvironment in cancer biology, the prognostic significance of related biomarkers has been investigated in DLBCL. PLT, an important host factor, contributed to tumor cell proliferation and metastatic progression [43–45]. Previous investigations showed that thrombocytosis was significantly associated with poor survival in a variety of solid tumors, including non-small cell lung cancer [46], gastric cancer [47] and ovarian cancer [48]. Contrarily, there are relatively few reports focusing on the role of PLT in predicting outcomes for lymphoma. In contradiction with solid tumors, several studies involving DLBCL reported that thrombocytopenia had an adverse impact on survival outcomes [49, 50]. The current study also confirmed the previous results that low PLT level was adversely associated with both OS and PFS. The explanation for these distinct observations remains unclear. Interestingly, two early studies demonstrated that thrombocytopenia adversely affected survival only among lymphoma patients with BM involvement [51, 52]. However, our previous study demonstrated that the low platelet count was significantly predictive of survival in patients with or without BM involvement [53]. Besides, our current study showed low PLT level was an independent poor prognostic marker in DLBCL after adjusting for BM involvement. Therefore, it remains ambiguous whether the predictive significance of thrombocytopenia was attributable to the BM involvement. Another important issue which should be considered was that our study did not imply thrombocytosis was not associated with inferior prognosis. One possibility was that the relationship between the platelet count and prognosis might be not necessarily linear, but might be U-shaped. However, the platelet count could only be divided into dichotomous variables with the Maximally Selected Rank Statistics in our current study, and only one cutoff value that would provide the best separation of the survival outcomes into two groups was identified. Therefore, whether thrombocytosis was also predictive of prognosis in DLBCL were not assessed in our study. Given these findings, further in-depth analyses of platelets in patients with DLBCL are required to fully understand the prognostic role of platelets.

RDW, a simple and easily available index reflecting the variability in size of circulating erythrocytes, was proved to be a powerful prognostic marker in cardiovascular and thrombotic disorders [54, 55]. Also, several studies have evaluated the association between RDW and cancer, including solid tumors and hematological cancer, and suggested that increased RDW was correlated with advanced stage and worse prognosis [56, 57]. In a study involving 81 patients with DLBCL, patients with RDW > 15% had significantly worse survival outcomes compared with those with RDW ≤  15% [58]. Bento et al. also found that high RDW level predicted an unfavorable PFS and OS, adding prognostic information in patients with DLBCL [16]. The biologic mechanisms underlying this association are not fully understood, though some data suggested the correlation of RDW level with systemic inflammatory state, nutritional deficiency and oxidative stress which were, actually, important risk factors for cancer [59, 60]. In agreement with these observations, our study also confirmed the evidence of a statistically significant association between high RDW level and inferior prognosis.

In the present study, the proposed new prognostic model incorporated IPI and three easily available variables, including β2M, PLT and RDW. The new model performed well in predicting OS. The new prognostic model identified a very favorable prognostic group with the 5-year OS rate of approximately 90%. For this subgroup, the standard R-CHOP regimens may be enough to exhibit excellent outcomes. Meanwhile, patients falling into the very high-risk group had a 5-year OS rate of less than 30%, which should be considered in clinical studies for more aggressive induction therapy, or additional consolidation therapy, or innovative treatment approaches. When compared to the IPI, R-IPI and NCCN-IPI, the new model displayed a superior performance in both training and validation cohorts. Indeed, compared with the IPI, the new model improved the ability to identify a subset of patients with more favorable survival, and also captured more patients at high risk for disease progression and death. Also, the new model retained the ability of the R-IPI to identify the very-good risk group, while outperforming the R-IPI by enhancing identification of high-risk disease. Also, consistent with previous reports [7, 10, 61], we found that the NCCN-IPI well distinguished a very poor-risk group, whereas only a small minority of patients could be classified as this risk category. These findings imply that our new prognostic model that considers tumor-bearing host features and tumor microenvironment could provide additional prognostic information than conventional models. It is of great importance to add these variables to traditional patient- or tumor-specific features. However, since our new model incorporated β2M, PLT, RDW in conjunction with the IPI, it was obviously more cumbersome than the IPI or R-IPI in calculating the score in clinical practice. Despite this, all of the variables included in the new model were easily attainable and obviously reproducible in real-life practice. Besides, this new model was built on the basis of a large database of patients treated with standard R-CHOP regimens, which may be applicable to the current treatment era. Taken together, after validation in an independent cohort, the new model proposed in this study might provide a reliable and useful tool for predicting outcome for DLBCL patients treated with R-CHOP regimens, aiding in the development of risk-adapted treatment approaches.

Also, our study has several important limitations. First, due to the retrospective nature of this study, a small proportion of patients with missing data on one or more clinical features were deleted from this study. However, these were missing at random, which therefore should not lead to an obvious bias. Second, our data was obtained from a single center in China, so it is still unclear whether the new prognostic model could be applicable to other centers. Further validation of the new prognostic model in independent series is warranted. Besides, since this study was retrospectively conducted, and most patients were diagnosed prior to the reclassification of patients with *MYC* and *BCL2* or/and *BCL6* rearrangements (the so-called double-hit lymphoma [DHL] or triple-hit lymphoma [THL]) as a new category in the high-grade B-cell lymphoma in 2016 [62], the information on the proportion of patients with DHL or THL was unavailable. Therefore, the ability of the new prognostic model to identify patients with poor prognosis remains unclear when those with DHL or THL were excluded. Finally, this study was based solely on clinical data, and the ability of the new model to identify a very high-risk group remains somewhat disappointing, with only roughly 13% of patients stratified into the very high-risk group exhibiting 5-year OS of below 30%. The optimization of the new model by adding novel factors, such as pathological or biologic markers with prognostic significance, might further improve the accuracy.

## Conclusion

In conclusion, the new prognostic model as proposed in this study might be a useful predictive tool for DLBCL patients treated with R-CHOP regimens. However, the prognostic significance of this new model should be validated in independent series or in prospective cohorts. Once our findings have been validated, the identification of low or high risk groups by this new prognostic model will potentially guide the design of future clinical studies. Those low-risk patients may achieve cure with the current standard R-CHOP regimen. Conversely, those high-risk groups may benefit from alternative intensified treatment or novel therapeutic approaches.

## Supplementary Information

**Additional file 1: Table S1.** Univariate analyses for progression-free survival and overall survival in the training cohort**. Table S2.** Multivariate analyses for progression-free survival and overall survival in the training cohort. **Table S3.** The Harrell’s C-index for 5-year overall survival prediction. **Table S4.** The association of serum creatinine level and serum β2M level in all patients. **Fig. S1** Kaplan–Meier curves of survival outcomes in the training cohort. **Fig. S2** Calibration curves. **Fig. S3** Progression-free survival (PFS) for risk groups defined by four prognostic models in the training cohort. **Fig. S4** Progression-free survival (PFS) for risk groups defined by four prognostic models in the validation cohort.

## Data Availability

The datasets generated and/or analysed during the current study are not publicly available due to that the data also forms part of other ongoing studies but are available from the corresponding author on reasonable request.

## Abbreviations

CHOP: Cyclophosphamide, doxorubicin, vincristine, prednisone
NHL: Non-Hodgkin’s lymphoma
DLBCL: Diffuse large B-cell lymphoma
IPI: International Prognostic Index
R-IPI: Revised International Prognostic Index
NCCN-IPI: Enhanced National Comprehensive Cancer Network International Prognostic Index
β2M: β2-microglobulin
R-CDOP: Rituximab, cyclophosphamide, pegylated liposomal doxorubicin, vincristine, prednisone
R-CHOPE: Rituximab, cyclophosphamide, doxorubicin, vincristine, prednisone, etoposide
ECOG: Eastern Cooperative Oncology Group
PS: Performance status
LDH: Lactate dehydrogenase
BM: Bone marrow
CBC: Complete blood count
ALC: Absolute lymphocyte count
AMC: Absolute monocyte count
ANC: Absolute neutrophil count
PLT: Platelet
RDW: Red blood cell distribution width
PDW: Platelet distribution width
MPV: Mean platelet volume
LMR: The lymphocyte to monocyte ratio
NLR: The neutrophil to lymphocyte ratio
PLR: The platelet to lymphocyte ratio
OS: Overall survival
PFS: Progression-free survival
C-index: Concordance index
ROC: Receiver operating characteristic
AUC: Area under curve
IBS: Integrated Brier score
DCA: Decision curve analysis
HR: Hazard ratio
