# Supplementary material for: Enhancement of the International prognostic index with β2-microglobulin, platelet count and red blood cell distribution width: a new prognostic model for diffuse large B-cell lymphoma in the rituximab era
Source: BMC Cancer. 2022 May 27;22:583. doi: 10.1186/s12885-022-09693-z (PMC9137167; doi:10.1186/s12885-022-09693-z)

**Supplementary Material**

**Enhancement of the International Prognostic Index with β2-microglobulin, platelet count and red blood cell distribution width: a new prognostic model for diffuse large B-cell lymphoma in the rituximab era**

Table of Content

[Table S1. Univariate analyses for progression-free survival and overall survival in the training cohort 2](#_Toc101544142)

[Table S2. Multivariate analyses for progression-free survival and overall survival in the training cohort 3](#_Toc101544143)

[Table S3. The Harrell’s C-index for 5-year overall survival prediction 4](#_Toc101544144)

[Table S4 The association of serum creatinine level and serum β2M level in all patients 5](#_Toc101544145)

[Fig. S1 Kaplan–Meier curves of survival outcomes in the training cohort. 6](#_Toc101544146)

[Fig. S2 Calibration curves. 7](#_Toc101544147)

[Fig. S3 Progression-free survival (PFS) for risk groups defined by four prognostic models in the training cohort. 8](#_Toc101544148)

[Fig. S4 Progression-free survival (PFS) for risk groups defined by four prognostic models in the validation cohort. 9](#_Toc101544149)

# Table S1. Univariate analyses for progression-free survival and overall survival in the training cohort

| Prognostic factors | Progression-free survival | | Overall survival | |
| --- | --- | --- | --- | --- |
|  | HR (95%CI) | *P* value | HR (95%CI) | *P* value |
| Age >60 years | 1.442 (1.131-1.838) | 0.003 | 1.738 (1.322-2.285) | <0.001 |
| Male gender | 1.012 (0.897-1.141) | 0.847 | 1.017 (0.887-1.167) | 0.807 |
| ECOG PS ≥2 | 3.059 (2.266-4.129) | <0.001 | 3.991 (2.906-5.482) | <0.001 |
| Ann Arbor stage III-IV | 4.467 (3.466-5.755) | <0.001 | 4.187 (3.136-5.588) | <0.001 |
| Extranodal site ≥2 | 3.209 (2.513-4.098) | <0.001 | 3.287 (2.494-4.332) | <0.001 |
| Bone marrow involvement | 2.342 (1.572-3.487) | <0.001 | 2.286 (1.481-3.527) | <0.001 |
| LDH level > normal | 2.242 (1.751-2.865) | <0.001 | 2.732 (2.049-3.650) | <0.001 |
| β2M level > normal | 2.698 (2.121-3.431) | <0.001 | 2.815 (2.141-3.701) | <0.001 |
| Serum creatinine level > normal | 3.072 (1.824-5.175) | <0.001 | 3.039 (1.695-5.449) | <0.001 |
| Hemoglobin <120g/L | 1.883 (1.451-2.445) | <0.001 | 1.961 (1.460-2.632) | <0.001 |
| Albumin <35g/L | 2.488 (1.742-3.546) | <0.001 | 2.284 (1.514-3.445) | <0.001 |
| ALC <1.75×10^9^/L | 1.431 (1.112-1.838) | 0.005 | 1.917 (1.417-2.592) | <0.001 |
| AMC ≥0.65×10^9^/L | 1.946 (1.509-2.510) | <0.001 | 1.951 (1.462-2.602) | <0.001 |
| ANC ≥6.41×10^9^/L | 1.891 (1.405-2.544) | <0.001 | 1.887 (1.348-2.642) | <0.001 |
| PLT <157×10^9^/L | 2.093 (1.558-2.811) | <0.001 | 2.305 (1.668-3.184) | <0.001 |
| LMR <2.55 | 2.467 (1.940-3.138) | <0.001 | 2.737 (2.083-3.596) | <0.001 |
| NLR ≥3.68 | 2.009 (1.564-2.581) | <0.001 | 2.113 (1.593-2.802) | <0.001 |
| PLR ≥184 | 1.616 (1.268-2.060) | <0.001 | 1.872 (1.423-2.463) | <0.001 |
| RDW ≥14.5% | 2.466 (1.849-3.288) | <0.001 | 3.015 (2.209-4.114) | <0.001 |
| PDW ≥12.8 fl | 1.255 (0.969-1.626) | 0.085 | 1.349 (1.010-1.803) | 0.043 |
| MPV <9.1 fl | 1.154 (0.833-1.600) | 0.388 | 1.332 (0.930-1.908) | 0.134 |
| IPI risk group |  |  |  |  |
| Low (0-1) | Reference |  | Reference |  |
| Low-intermediate (2) | 2.548 (1.841-3.597) | <0.001 | 3.342 (2.265-4.931) | <0.001 |
| High-intermediate (3) | 4.519 (3.311-6.168) | <0.001 | 5.194 (3.584-7.526) | <0.001 |
| High (4-5) | 7.483 (5.251-10.664) | <0.001 | 9.891 (6.628-14.762) | <0.001 |

**Abbreviation:** ECOG, Eastern Cooperative Oncology Group; PS, performance status; LDH, lactate dehydrogenase; β2M, β2-microglobulin; ALC, absolute lymphocyte count; AMC, absolute monocyte count; ANC, absolute neutrophil count; PLT, platelet; LMR, the lymphocyte to monocyte ratio; NLR, the neutrophil to lymphocyte ratio; PLR, the platelet to lymphocyte ratio; RDW, red blood cell distribution width; PDW, platelet distribution width; MPV, mean platelet volume; IPI, International Prognostic Index; HR, hazard ratio; CI, confidence interval

# Table S2. Multivariate analyses for progression-free survival and overall survival in the training cohort

| Prognostic factors | Progression-free survival | | Overall survival | |
| --- | --- | --- | --- | --- |
|  | HR (95%CI) | *P* value | HR (95%CI) | *P* value |
| Bone marrow involvement | 1.445 (0.951-2.193) | 0.085 | 1.119 (0.703-1.780) | 0.636 |
| β2M level > normal | 1.543 (1.181-2.016) | 0.001 | 1.411 (1.040-1.913) | 0.027 |
| Serum creatinine level > normal | 1.497 (0.839-2.673) | 0.172 | 1.517 (0.790-2.912) | 0.211 |
| Hemoglobin <120g/L | 0.873 (0.628-1.214) | 0.420 | 0.846 (0.588-1.217) | 0.366 |
| Albumin <35g/L | 1.245 (0.822-1.885) | 0.302 | 1.007 (0.633-1.604) | 0.976 |
| ALC <1.75×10^9^/L | 0.859 (0.621-1.188) | 0.357 | 1.082 (0.739-1.583) | 0.686 |
| AMC ≥0.65×10^9^/L | 1.033 (0.757-1.410) | 0.837 | 1.053 (0.737-1.505) | 0.776 |
| ANC ≥6.41×10^9^/L | 1.058 (0.729-1.534) | 0.768 | 1.169 (0.767-1.780) | 0.468 |
| PLT <157×10^9^/L | 1.433 (1.010-2.034) | 0.044 | 1.548 (1.038-2.308) | 0.032 |
| LMR <2.55 | 1.293 (0.917-1.824) | 0.143 | 1.295 (0.874-1.920) | 0.198 |
| NLR ≥3.68 | 1.135 (0.771-1.669) | 0.522 | 1.096 (0.710-1.692) | 0.678 |
| PLR ≥184 | 1.272 (0.886-1.828) | 0.192 | 1.477 (0.990-2.204) | 0.056 |
| RDW ≥14.5% | 1.438 (1.022-2.023) | 0.037 | 1.758 (1.214-2.547) | 0.003 |
| PDW ≥12.8 fl | ND | ND | 1.123 (0.815-1.548) | 0.479 |
| IPI risk group |  |  |  |  |
| Low (0-1) | Reference |  | Reference |  |
| Low-intermediate (2) | 2.003 (1.411-2.845) | <0.001 | 2.401 (1.593-3.618) | <0.001 |
| High-intermediate (3) | 2.922 (2.053-4.159) | <0.001 | 3.346 (2.211-5.064) | <0.001 |
| High (4-5) | 4.073 (2.684-6.182) | <0.001 | 5.341 (3.301-8.644) | <0.001 |

**Abbreviation:** β2M, β2-microglobulin; ALC, absolute lymphocyte count; AMC, absolute monocyte count; ANC, absolute neutrophil count; PLT, platelet; LMR, the lymphocyte to monocyte ratio; NLR, the neutrophil to lymphocyte ratio; PLR, the platelet to lymphocyte ratio; RDW, red blood cell distribution width; PDW, platelet distribution width; IPI, International Prognostic Index; HR, hazard ratio; CI, confidence interval; ND, not done.

# Table S3. The Harrell’s C-index for 5-year overall survival prediction

| Risk model | C-index (95%CI) | |
| --- | --- | --- |
|  | Training cohort | Validation cohort |
| New model | 0.750 (0.719-0.781) | 0.733 (0.682-0.784) |
| IPI | 0.723 (0.690-0.756) | 0.706 (0.653-0.759) |
| R-IPI | 0.710 (0.681-0.739) | 0.664 (0.610-0.718) |
| NCCN-IPI | 0.713 (0.682-0.744) | 0.664 (0.608-0.720) |

**Abbreviation:** C-index, concordance index; CI, confidence interval; IPI, International Prognostic Index; R-IPI, revised International Prognostic Index; NCCN-IPI, National Comprehensive Cancer Network International Prognostic Index

Table S4 The association of serum creatinine level and serum β2M level in all patients

|  | Overall, n (%) | Serum creatinine level | | |
| --- | --- | --- | --- | --- |
|  |  | Elevated, n (%) | Normal, (%) | *P* value |
| Serum β2M level |  |  |  |  |
| Elevated, n (%) | 310 (31.1) | 21 (75.0) | 289 (29.8) | <0.001 |
| Normal, n (%) | 688 (68.9) | 7 (25.0) | 681 (70.2) |  |

**Abbreviation:** β2M, β2-microglobulin

Fig. S1 Kaplan–Meier curves of survival outcomes in the training cohort. (a) Progression-free survival (PFS) according to β2-microglobulin (β2M); (b) Overall survival (OS) according to β2M; (c) PFS according to red blood cell distribution width (RDW); (d) OS according to RDW; (e) PFS according to platelet (PLT); (f) OS according to PLT.


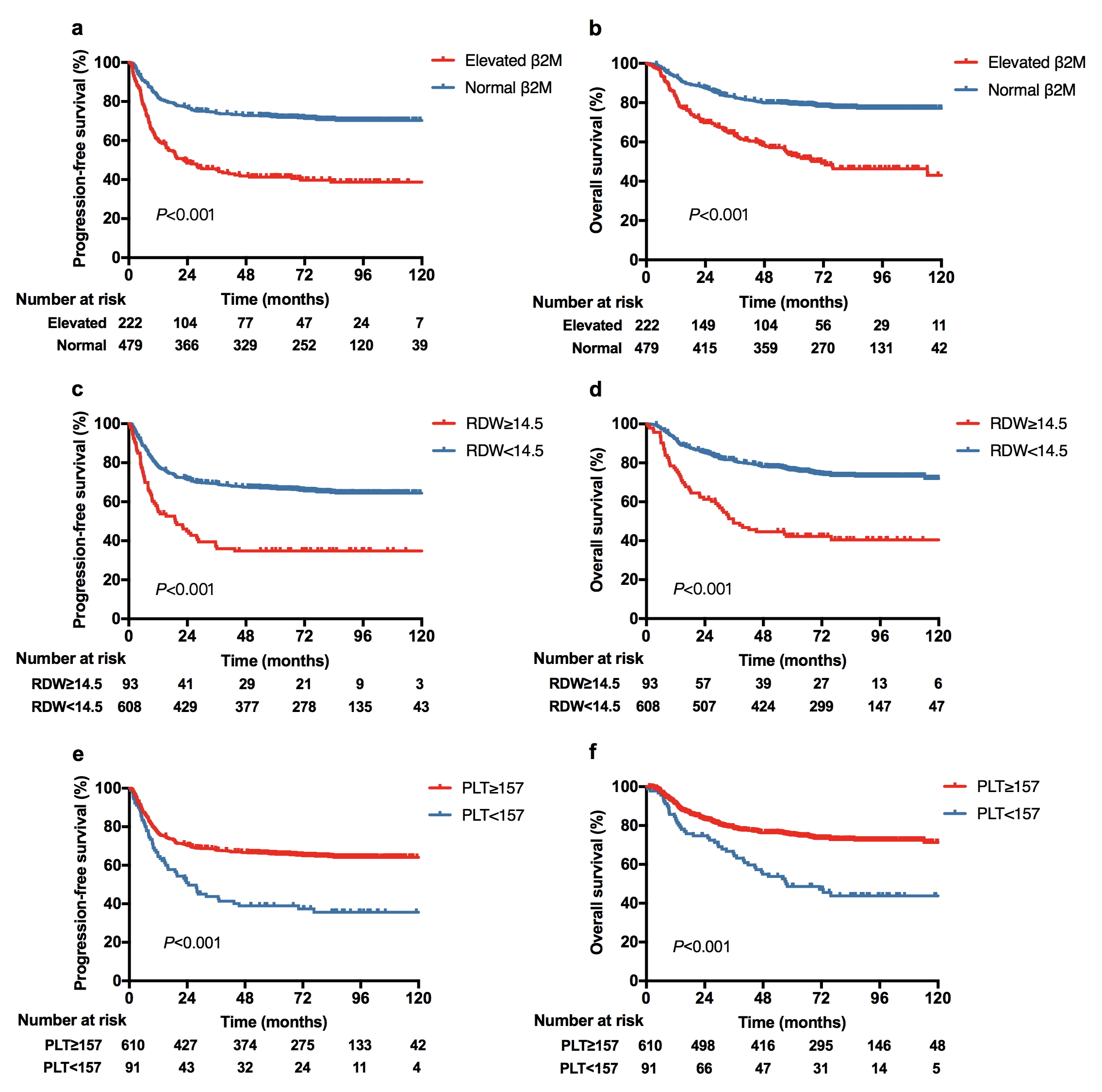


Fig. S2 Calibration curves. (a)The calibration curves for predicting 5-year overall survival (OS) in the training cohort; (b) The calibration curves for predicting 5-year OS in the validation cohort. The predicted OS by the new prognostic model is plotted on the x-axis, and the actual OS is plotted on the y-axis.


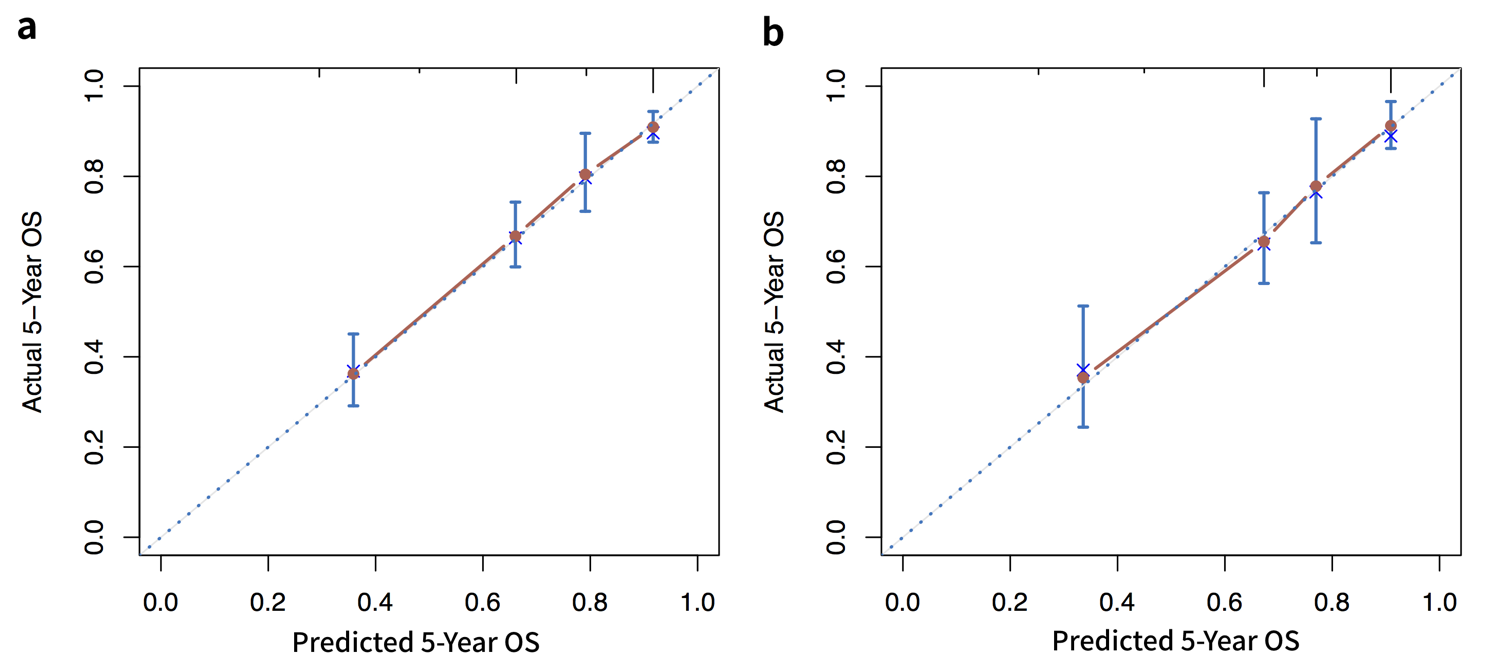


Fig. S3 Progression-free survival (PFS) for risk groups defined by four prognostic models in the training cohort. (a) PFS stratified by the new model; (b) PFS stratified by the International Prognostic Index (IPI); (c) PFS stratified by the revised IPI (R-IPI); (d) PFS stratified by the National Comprehensive Cancer Network IPI (NCCN-IPI).


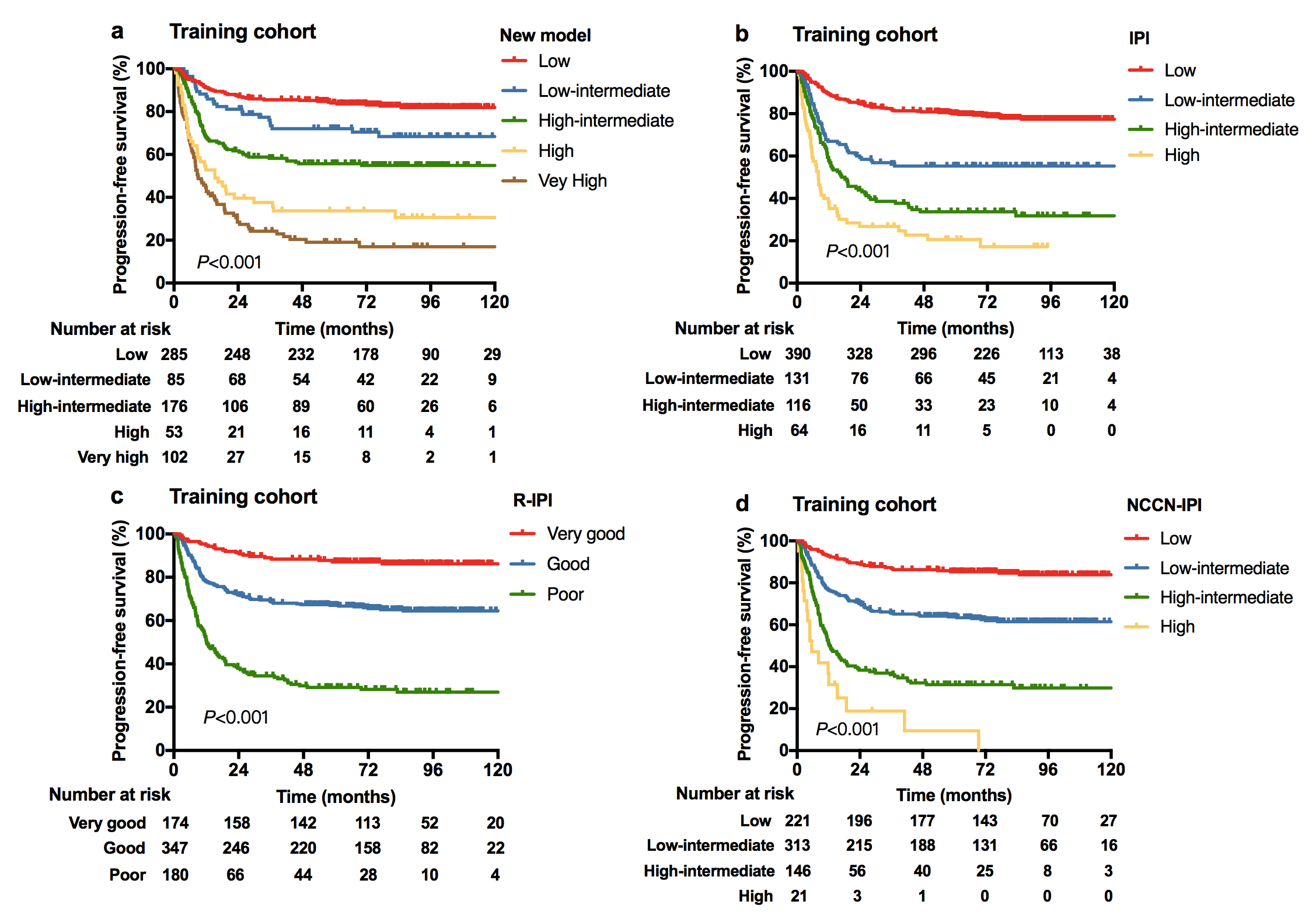


Fig. S4 Progression-free survival (PFS) for risk groups defined by four prognostic models in the validation cohort. (a) PFS stratified by the new model, (b) PFS stratified by the International Prognostic Index (IPI); (c) PFS stratified by the revised IPI (R-IPI); (d) PFS stratified by the National Comprehensive Cancer Network IPI (NCCN-IPI).


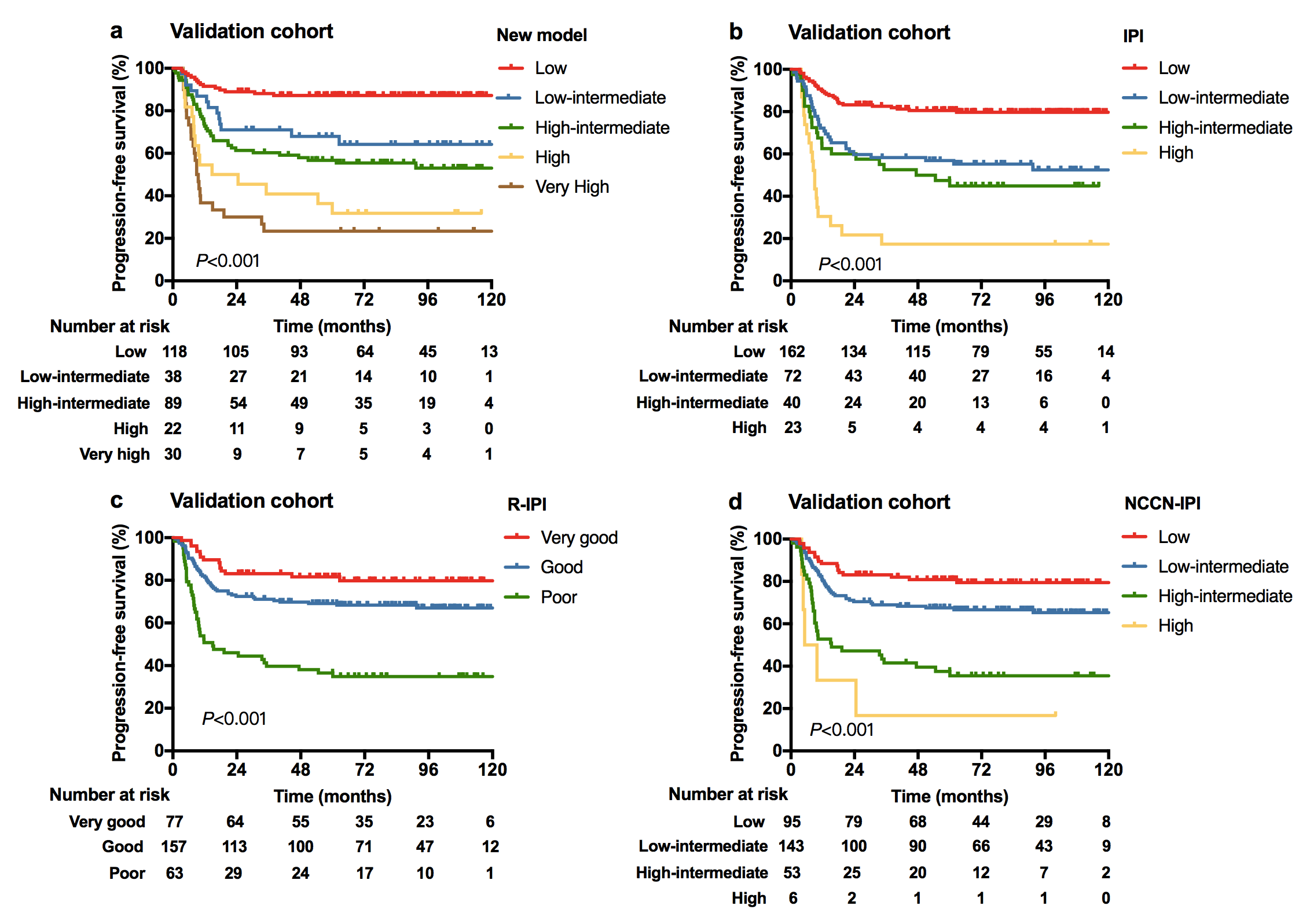

Supplement: Supplementary file 1 — Additional file 1: Table S1. Univariate analyses for progression-free survival and overall survival in the training cohort. Table S2. Multivariate analyses for progression-free survival and overall survival in the training cohort. Table S3. The Harrell’s C-index for 5-year overall survival prediction. Table S4. The association of serum creatinine level and serum β2M level in all patients. Fig. S1 Kaplan–Meier curves of survival outcomes in the training cohort. Fig. S2 Calibration curves. Fig. S3 Progression-free survival (PFS) for risk groups defined by four prognostic models in the training cohort. Fig. S4 Progression-free survival (PFS) for risk groups defined by four prognostic models in the validation cohort. [file 12885_2022_9693_MOESM1_ESM.docx]
